# Interactions between the cortical midline structures and sensorimotor network track maladaptive self-beliefs in clinical high risk for psychosis

**DOI:** 10.1038/s41537-022-00279-z

**Published:** 2022-09-16

**Authors:** Henry R. Cowan, Katherine S. F. Damme, Vijay A. Mittal

**Affiliations:** 1 Psychology, Northwestern University, Evanston, IL USA; 2 Psychology, Psychiatry, Medical Social Sciences, Institute for Policy Research, Northwestern University, Evanston, IL USA

**Keywords:** Psychosis, Neural circuits, Biomarkers

## Abstract

Individuals at clinical high risk for psychosis (CHR) report a maladaptive self-concept—with more negative and less positive self-beliefs—linked to clinical symptoms and functional impairment. Alterations have also been reported in brain networks associated with intrinsic (cortical midline structures, CMS) and extrinsic (sensorimotor network, SMN) self-processing. Theoretical accounts of multiple levels of self-experience in schizophrenia suggest that interactions between these networks would be relevant for self-beliefs. This study tested whether self-beliefs related to resting-state functional connectivity within and between the CMS and SMN. Participants were 56 individuals meeting CHR criteria and 59 matched healthy community participants (HC). Pearson correlations examined potential mediators and outcomes. The CHR group reported more negative and less positive self-beliefs. Greater resting-state functional connectivity between the posterior CMS (posterior cingulate cortex) and the SMN was associated with less positive self-beliefs in CHR, but more positive self-beliefs in HC. Attenuated negative symptoms and poorer social functioning were associated with CMS-SMN connectivity (trend level after FDR-correction) and self-beliefs. Reduced connectivity between the left and right PCC was associated with lower positive self-beliefs in CHR, although this effect was specific to very low levels of positive self-beliefs. Left-right PCC connectivity did not correlate with outcomes. Dynamic interactions between intrinsic and extrinsic self-processing supported positive self-beliefs in typically developing youth while undermining positive self-beliefs in CHR youth. Implications are discussed for basic self-fragmentation, narrative self-related metacognition, and global belief updating. Interventions for self-processing may be beneficial in the CHR syndrome.

## Introduction

Individuals with schizophrenia tend to hold unusually strong negative beliefs and few positive beliefs about themselves, forming a maladaptive self-concept with low global self-esteem^1,2^ and cognitive feedback loops which maintain psychotic symptoms^3,4^. The same pattern of maladaptive self-concept, with more negative and less positive self-beliefs, has been observed in the clinical high risk for psychosis syndrome (CHR), a high risk state that often predates the onset of psychotic disorders^5–7^. Crucially, longitudinal studies have found that self-beliefs track the pathogenic mechanisms leading toward psychotic disorders: in CHR samples, self-beliefs become increasingly maladaptive over time for individuals who experience worsening symptoms^8^ or convert to a psychotic disorder^7^. The specific processes associated with dysfunctional self-concept in the CHR syndrome are therefore likely to inform the overall progression of psychotic disorders and suggest new avenues for intervention in this critical population. However, the complex multilevel structure of self-experience, together with impaired cognitive insight^9^ and self-disturbances^10^ in the CHR syndrome, may limit the utility of self-report or introspection to access mechanisms underlying self-beliefs in this population.

Resting-state functional connectivity between relevant brain networks can shed light on neural mechanisms underlying self-beliefs, independent of individuals’ explicit insight into these processes^11^. Self-disturbances have been observed in psychotic disorders affecting two distinct levels of the self, with links to relevant brain networks. The basic or minimal self (i.e., the prereflective experience of subjectivity in the present moment) is fundamentally altered in psychotic disorders, chiefly in a “heightened awareness of aspects of experience that are normally tacit or implicit” and “a weakened sense of existing as a subject of awareness”^10,12,13^. Similar basic self-disturbances have been observed in the CHR syndrome^10^. The narrative or autobiographical self, by contrast, is a temporally extended consciousness of oneself as an object of experience—as an individual with traits, goals, values, social roles, and so on^13,14^. Disruptions in the narrative self have been observed in psychotic disorders from various perspectives including metacognitive self-awareness^15,16^, dialogical or dialectical self-awareness (i.e., self-understanding based on one’s position in the physical, social, and cultural environment)^16,17^, lifespan identity development^18^, and narrative identity^19^. Self-beliefs would most likely be located at the narrative level of self, as self-schemas^20,21^ or “meta-positions”^17^ representing one’s characteristic ways of being in the world.

Both levels of self entail some amount of integration between internal cognitive-affective experiences and interactions with the external world. The basic self is most commonly associated with processing of internal self-referential information (e.g., self-related thoughts and emotions)^22^. However, the basic self is “most of the time an acting self” engaged in real or imagined movement, perception, and proprioception^23,24^ embedded in a “self-world structure” in which self-experience is juxtaposed against the backdrop of a reliable, predictable external world^13,25^. Integration of intrinsic and extrinsic processing supports the normative functioning of the basic self, and fragmentation of intrinsic and extrinsic processing may underlie the self-disturbances observed in schizophrenia^11^. Similarly, the narrative self is built on the foundations of the basic self^13^, and is theorized to emerge over time as basic self-experience co-occurs with encoding and retrieval of autobiographical memories^14,21,26^. Therefore, narrative self-experience should also entail integration of intrinsic and extrinsic processing.

And in fact, narrative self-experience probably involves even more intrinsic-extrinsic integration than does basic self-experience. The primary ingredients of the narrative self are socially constructed autobiographical memories that define “the self, other people, and typical interactions with others and the surrounding world…drawn largely from the influences of familial and peer socialization, schooling, and religion, as well as [culture]”^21^. These ingredients are particularly relevant in the CHR age range, a developmental period in which youth form the initial autobiographical memories on which self-beliefs are based^27,28^. Empirically, personally-relevant memories in middle-late adolescence (ages 14–18) focus on academic and sports performance, physical appearance, grooming, and adherence to social norms^28^, changing focus to career choices, educational attainment, and dating relationships in emerging adulthood (ages 18–25)^29,30^. These memory categories plainly entail interactions with others in the external world.

Moreover, the metacognitive processes that assemble autobiographical memories into a narrative self are also thought to be intersubjective and dialectical. These processes first appear through dialogue, in conversations with caregivers in early childhood^31^. Self-referential metacognition then develops “via intersubjectivity, either formed explicitly with and in the company of others or in the context of implied or imagined others”^32^. Metacognition evolves into an awareness of multiple ways in which the self is positioned relative to others and the world^17^. This awareness then supports a sense of personal agency as individuals balance multiple self-positions to organize cognition, emotion, and behavior toward coherent personal goals^17,21^. The metacognitive processes that form a narrative self from basic self-experience and autobiographical memory would thus seem to rely heavily on integration of intrinsic and extrinsic self-processing.

In the brain, self-referential processing of internal cognitive-affective experiences is most commonly associated with the *cortical midline structures* (CMS), a circuit comprising major components of the default mode network including the medial prefrontal cortex (mPFC), anterior cingulate cortex (ACC), and posterior cingulate cortex (PCC)^33,34^. Processing of real or imagined interactions with the external environment is most associated with the sensorimotor network (SMN), centered on the precentral gyrus (primary motor cortex) and postcentral gyrus (primary sensory cortex)^11,24,35,36^. Interactions between the CMS and SMN, passing through hub regions including the posterior cingulate cortex^37^, support a healthy integration of intrinsic- and extrinsic self-processing^11^. In schizophrenia, the CMS are typically found to be elevated in activity and connectivity at rest relative to control samples^38–42^, with a lack of normative task-based suppression^43^. These patterns relate to clinical symptoms of schizophrenia including hallucinations^44–46^ and delusions^47,48^, as well as associated features including aberrant salience^49^, faulty emotional appraisals^47^, impaired insight^50,51^, and cognitive impairment^43^. Similarly, SMN hypoactivity has been observed in schizophrenia and has been linked to global hallucination severity^52^ as well as specific experiences of alien control^53,54^, thought insertion^54^, and auditory hallucinations^55^. Finally, interactions between the CMS and SMN are disrupted in schizophrenia^56–58^. Functional isolation of the two networks and reduced network modularity have been theorized to result in self-fragmentation (i.e., failure to integrate intrinsic and extrinsic self-processing), which in turn contributes to positive and negative symptoms, impaired insight, and interpersonal problems^11^.

Resting-state functional connectivity within and between the CMS and SMN appears to be disrupted in the CHR syndrome as well, although fewer studies have examined these processes in CHR. Several CHR studies have reported increased CMS activation, increased CMS connectivity, and attenuated CMS task-based suppression, similar to common findings in schizophrenia^22,59–61^. Increased CMS resting-state connectivity has been linked directly to impaired clinical insight^59^, while lack of CMS task-based suppression has been linked to reality distortion and impaired cognition^61^. Connectivity patterns may relate to other alterations in intrinsic self-processing observed behaviorally in CHR, including in self-referential reasoning biases^62^, impaired emotion awareness^63^, and heightened self-certainty^9^. The SMN was also found to have reduced connectivity at rest in one CHR sample^64^, which may relate to deficits observed behaviorally in motor coordination^65–67^, motor agency^68^, dyskinesia^65,69^, and sensorimotor gating^70–72^. To date, interactions between the CMS and SMN have not been studied in the CHR literature.

The available evidence in schizophrenia and the CHR syndrome suggests that dysfunction in the self-concept should relate to dysconnectivity between the CMS (reflecting altered intrinsic self-processing) and the SMN (reflecting altered extrinsic self-processing). The current study aimed to test these relationships. Comparing a group meeting CHR criteria against a matched healthy comparison group, we hypothesized that a maladaptive self-concept in the CHR group would relate to: (a) increased connectivity within the CMS, reflecting increased or inefficient intrinsic self-processing; (b) reduced connectivity within the SMN, reflecting diminished extrinsic self-processing; and/or (c) altered connectivity between the CMS and SMN, reflecting faulty integration of intrinsic and extrinsic self-processing. Finally, in post hoc analyses, the current study tested relationships between connectivity findings, potential mediators (perceptual abnormalities and motor disturbances), and relevant outcomes (attenuated positive symptoms, attenuated negative symptoms, and social functioning).

## Results

### Participant characteristics

CHR participants (*n* = 56) were 39 (69%) Caucasian, 11 (20%) Hispanic/Latin, 3 (5%) Asian, and 3 (5%) other; 7 (13%) left-handed; with a mean age of 18.66 (SD = 1.74); a mean of 12.49 years of education (SD = 1.67); and a median annual household income of $40,000–59,999. As is common in CHR samples, some participants were prescribed psychiatric medication. In the CHR group, 24 participants (43%) were prescribed psychiatric medication, of whom 13 (23%) were prescribed more than one class of medication. SSRIs and stimulants were most common (*n* = 9, 16% each), followed by other antidepressants and antipsychotics (*n* = 8, 14% each) and mood stabilizers (*n* = 6, 11%). HC participants (*n* = 59) were 36 (61%) Caucasian, 12 (20%) Hispanic/Latin, 8 (14%) Asian, and 3 (5%) other; 4 (7%) left-handed; with a mean age of 18.36 (SD = 2.50); a mean of 12.39 years of education (SD = 2.48); and a median annual household income of $40,000–59,999. There were no significant differences between CHR and HC groups on any demographic variables (race, handedness, age, education, or income), confirmed by *t*-tests and chi-squared tests.

### Group differences in self-beliefs

**Fig. 1:**
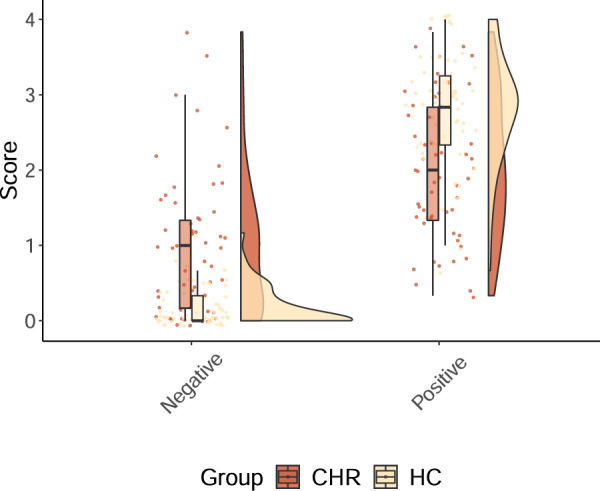
The clinical high risk for psychosis (CHR) group reported more negative and fewer positive self-beliefs than the healthy comparison (HC) group, as shown in this raincloud plot. Group means significantly differed with large effect sizes, negative *t*(64) = 6.16, *p* < 0.001, *d* = 1.16 [95% CI 0.75, 1.59], positive *t*(103) = −4.87, *p* < 0.001, *d* = −0.90 [95% CI −1.28, −0.51].

As shown in Fig. 1, CHR participants reported significantly more negative self-beliefs (mean = 0.92, SD = 0.85) than HC participants (mean = 0.19, SD = 0.26), *t*(64) = 6.16, *p* < 0.001, *d* = 1.16 [95% CI −1.59, −0.75]. CHR participants also reported significantly less positive self-beliefs (mean = 2.04, SD = 0.95) than HC participants (mean = 2.80, SD = 0.73), *t*(103) = −4.87, *p* < 0.001, *d* = −0.90 [95% CI −1.28, −0.51]. The distribution of negative self-beliefs suggests a likely floor effect in negative self-beliefs in the HC group.

### Resting-state functional connectivity

#### Group contrast

**Fig. 2:**
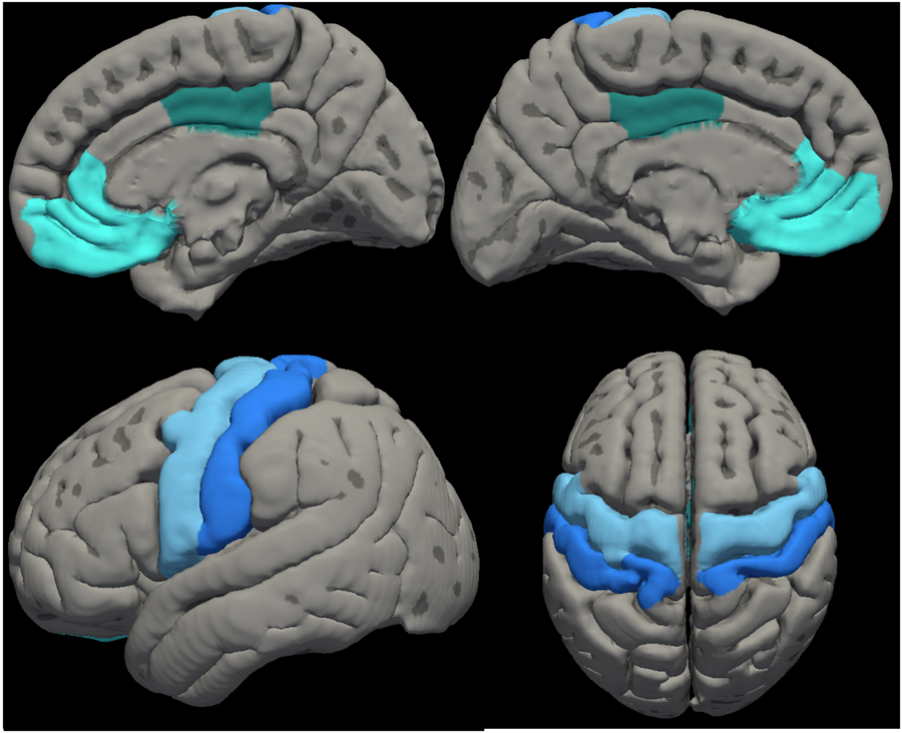
Regions of interest (ROIs) overlaid on the Desikan brain atlas^101^. CMS regions (in green) were the medial orbitofrontal and rostral anterior cingulate regions (combined into one medial prefrontal mask) and the posterior cingulate region. SMN regions (in blue) were the precentral region (primary motor cortex) and postcentral region (primary sensory cortex).

**Fig. 3:**
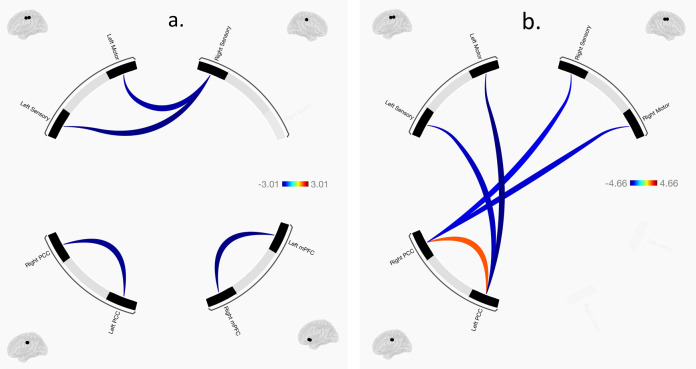
Significant functional connectivity results. **a** Group differences in functional connectivity (CHR>HC). Colors indicate t-statistics, with blue indicating negative effects (i.e., CHR has less connectivity than HC) and red indicating positive effects (i.e., CHR has more connectivity than HC). **b** Group differences in the relationship between positive self-beliefs and functional connectivity (i.e., group x positive self-beliefs interaction). Effects plotted for the group (CHR>HC) x positive self-beliefs interaction. Negative effects (in blue) indicate that the relationship between positive self-beliefs and connectivity is more negative in CHR than HC, while positive effects (in red) indicate that the relationship between positive self-beliefs and connectivity is more positive in CHR than HC. In both **a**, **b**, effects were thresholded at *p*_*FDR*_ < 0.05. Effects with *p*_*FDR*_ > 0.05 are omitted from this figure. No effects were significant for the negative self-beliefs × group interaction.

Regions of interest in the CMS and SMN are shown in Fig. 2 (see “Methods” below for more details). As shown in Table 1 and Fig. 3a, the CHR group showed a pattern of reduced interhemispheric connectivity within the CMS and SMN networks. Connectivity was reduced in CHR compared to HC between the left mPFC and right mPFC, *t*(55) = −3.01, *p*_FDR_ = 0.039; the left PCC and right PCC, *t*(55) = −2.97, *p*_FDR_ = 0.039; the left primary sensory cortex and right primary sensory cortex, *t*(55) = −2.92, *p*_FDR_ = 0.039; and the left primary motor cortex and right primary motor cortex, *t*(55) = −2.78, *p*_FDR_ = 0.044. These effects may reflect broad reductions in interhemispheric connectivity or specific reductions in connectivity within the CMS and SMN.

**Table 1:** Significant functional connectivity results.

Model	ROI–ROI connectivity	*t*	*p*_*FDR*_
Group contrast (CHR>HC)	L mPFC – R mPFC	−3.01	0.039
	L Sensory – R Sensory	−2.97	0.039
	L PCC – R PCC	−2.92	0.039
	L Motor – R Sensory	−2.78	0.044
Positive self-beliefs x group (CHR>HC) interaction	L PCC – L Motor	−4.66	<0.001
	L PCC – L Sensory	−4.06	0.001
	R PCC – R Motor	−3.91	0.001
	R PCC – R Sensory	−3.67	0.002
	L PCC – R PCC	2.75	0.039

ROIs shown here had significant connectivity at the ROI-to-ROI level (*p*_*FDR*_ < 0.05). No ROIs had significant connectivity in the negative self-beliefs x group interaction model. ROIs defined anatomically from the Desikan brain atlas.

*mPFC* medial prefrontal cortex, *PCC* posterior cingulate cortex, *Sensory* primary sensory cortex, *Motor* primary motor cortex.

#### Positive self-beliefs

**Fig. 4:**
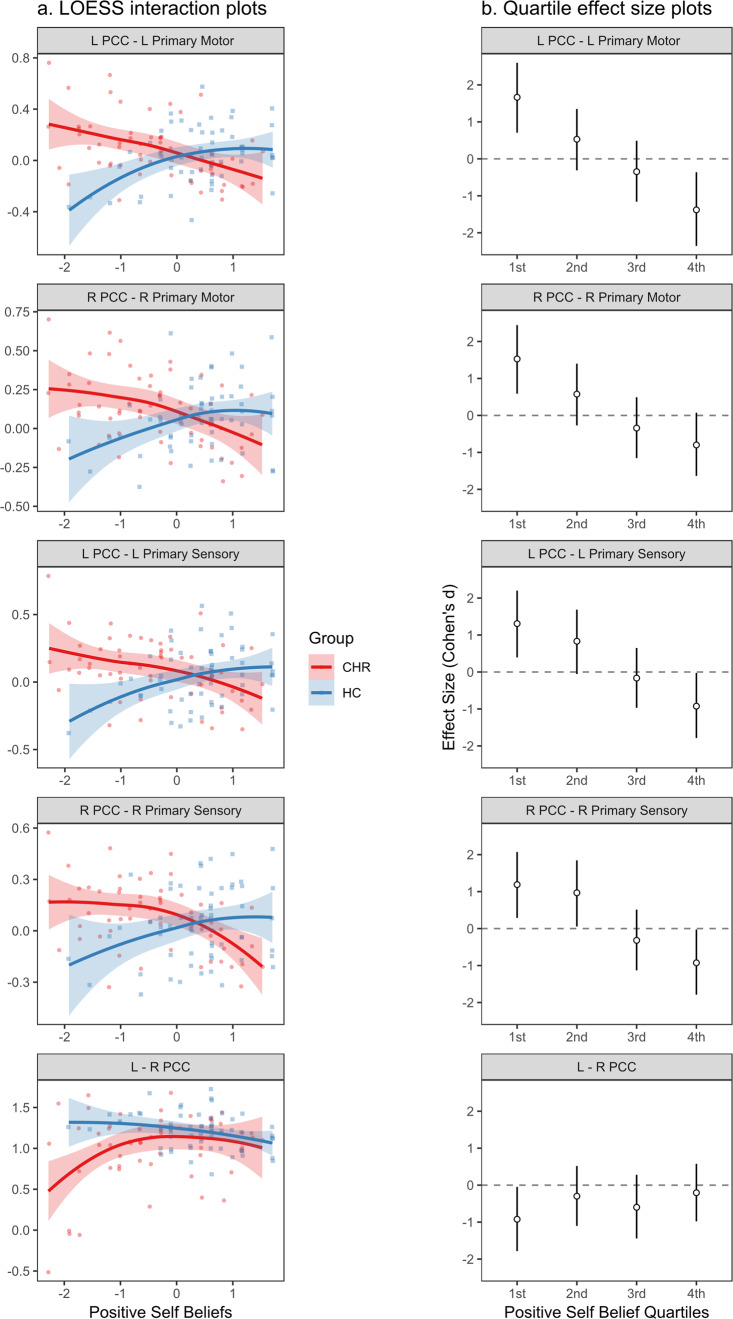
Interactions between group (CHR vs. HC) and positive self-beliefs in predicting connectivity. **a** Interactions plotted as LOESS (locally estimated slopes) regression lines to highlight any nonlinearity. Colored bands indicate the standard error of the LOESS regression lines. **b** Interactions plotted as effect sizes of mean differences in connectivity (CHR>HC) in each quartile of positive self-belief scores. Effect sizes expressed as Cohen’s *d*, with error lines indicating 95% confidence intervals.

As shown in Table 1, Figs. 3b, and 4, group (CHR>HC) interacted with positive self-beliefs in predicting functional connectivity between the left PCC and left primary motor area, HC *r* = 0.36, CHR *r* = −0.46, interaction *t*(113) = −4.66, *p*_*FDR*_ < 0.001, and between the left PCC and left primary sensory area, HC *r* = 0.34, CHR *r* = −0.38, interaction *t*(113) = −4.06, *p*_*FDR*_ = 0.001. A similar pattern was observed on the contralateral side, with self-beliefs predicting increased connectivity between the right PCC and right primary motor area, HC *r* = 0.28, CHR *r* = −0.42, interaction *t*(113) = −3.91, *p*_*FDR*_ = 0.001, and between the right PCC and right primary sensory area, HC *r* = 0.25, CHR *r* = −0.43, interaction *t*(113) = −3.67, *p*_*FDR*_ = 0.002. LOESS regression plots and quartile effect size plots (Fig. 4) indicated that these effects were consistent across the full range of positive self-beliefs. In sum, the groups showed opposite patterns: positive self-beliefs were associated with increased functional connectivity between CMS and SMN regions in the HC group, whereas they were associated with decreased functional connectivity between CMS and SMN regions in the CHR group.

Group (CHR>HC) also interacted with positive self-beliefs in predicting functional connectivity between the left and right PCC, HC *r* = −0.29, CHR *r* = 0.26, *t*(113) = 2.75, *p*_*FDR*_ = 0.039. LOESS regression plots and quartile effect size plots (Fig. 4) suggested that this effect was driven by large group differences when positive self-beliefs were very low.

Group did not interact with positive self-beliefs to predict functional connectivity between any other pairs of ROIs (right primary motor area – left primary motor area, right primary sensory area—left primary sensory area, right PCC—left primary motor area).

#### Negative self-beliefs

Group (CHR vs. HC) did not interact with negative self-beliefs in predicting functional connectivity between any pairs of ROIs.

#### External validators

As shown in Table 2, self-beliefs and connectivity scores did not correlate with potential mediators (perceptual abnormalities and motor disturbances, *r* ≤ |0.217|, *p*_*FDR*_ ≥ 0.180). With respect to outcome variables, positive self-beliefs correlated with attenuated negative symptoms (*r* = −0.560, *p*_*FDR*_ < 0.001) and social functioning (*r* = 0.429, *p*_*FDR*_ = 0.004). Mean PCC – SMN connectivity also correlated at FDR-corrected trend level with attenuated negative symptoms (*r* = 0.275, *p*_*FDR*_ = 0.094) and social functioning (*r* = −0.272, *p*_*FDR*_ = 0.094). Self-beliefs and connectivity scores did not correlate with positive symptoms. Left–right PCC connectivity did not correlate with any potential mediators or outcomes.

**Table 2:** Connectivity, self-beliefs, and external validators in the clinical high-risk group: FDR-corrected Pearson correlations.

	1	2	3	4	5	6	7
Connectivity and self-beliefs							
1. Mean PCC-SMN connectivity							
2. Left – right PCC connectivity	0.24						
3. Positive self-beliefs	−0.35*	0.26					
Sensorimotor disturbances							
4. Perceptual abnormalities	0.02	−0.07	−0.18				
5. Motor disturbances	0.12	−0.13	−0.22	0.02			
Symptoms and functioning							
6. Attenuated positive symptoms	0.08	−0.17	−0.19	0.65***	0.20		
7. Attenuated negative symptoms	0.27^†^	−0.14	−0.56***	0.29	0.51***	0.47**	
8. Social functioning	−0.27^†^	0.23	0.43**	−0.14	−0.41**	−0.42**	−0.72***

*PCC* posterior cingulate cortex, *SMN* sensorimotor network. Mean PCC-SMN connectivity score calculated as mean of connectivity between PCC and SMN regions of interest with significant group x self-beliefs interactions, as shown in Table 1 and Fig. 3b. ^†^*p*_*FDR*_ < 0.10; *p_*FDR*_ < 0.05; *p_*FDR*_ < 0.05; *p_*FDR*_ < 0.05.

## Discussion

This was the first study to test interactions between the CMS and SMN in the clinical high risk for psychosis syndrome. The current study linked low positive self-beliefs to abnormal resting-state functional connectivity between brain networks involved in intrinsic and extrinsic self-processing (the CMS and SMN, respectively). Consistent with theoretical accounts of intrinsic and extrinsic processing in the basic and narrative self, CHR and HC groups showed opposite associations between positive self-beliefs and CMS-SMN functional connectivity. In the HC group, increased functional connectivity between these networks was associated with more positive self-beliefs; by contrast, in the CHR group, increased functional connectivity between these networks was associated with less positive self-beliefs.

Developmentally, in adolescence and emerging adulthood, the self-concept is shaped through engagement with the environment^28–30^. In the HC group, positive self-beliefs were highest when the posterior CMS and SMN were more connected, suggesting that increased CMS-SMN connectivity normatively has a positive effect on the self-concept. In the CHR group, by contrast, positive self-beliefs were lowest when the posterior CMS and SMN were more connected, suggesting that increased CMS-SMN connectivity has a negative effect on the self-concept in CHR. Although a variety of sensorimotor issues have been observed in other CHR samples^65–69,71,72^, the present study’s results were not explained by the severity of perceptual abnormalities or motor disturbances, implying that the results do not simply reflect faulty sensorimotor processing. Rather, CMS-SMN connectivity and self-beliefs were associated with increased negative symptoms and social functional impairment, implying that integration of intrinsic and extrinsic self-processing may prompt negative self-evaluations associated with real or imagined experiences of anhedonia, avolition, or social failure. Correlations between CMS-SMN connectivity, negative symptoms, and social function reached trend-level after FDR-correction, with effect sizes in the conventional small-medium range^73^, whereas correlations between self-beliefs, negative symptoms, and social function reached significance after FDR-correction with effect sizes in the conventional medium-large range^73^. This pattern suggests that self-beliefs may be more proximal to outcomes, perhaps even mediating relationships between connectivity and outcomes. Future studies powered for mediation analysis^74^ within a CHR sample would be valuable to test this hypothesis.

An interesting implication of this finding is that self-referential metacognition may be somewhat aversive to individuals at CHR. This would be in line with previous research showing that autobiographical reflection is aversive for individuals with schizotypal traits^75^. A global deficit in updating prior beliefs based on new sensory data has been proposed as a common mechanism underlying the experience of psychosis at multiple levels of analysis^76,77^, including in CHR^78^. In schizophrenia, low positive self-beliefs are maintained in part by a lack of updating in response to belief-inconsistent information, e.g., positive feedback about the self^4,79^. Deficits in belief updating also predict which individuals with schizophrenia will respond to metacognitive training^80^, suggesting a causal link between belief updating and metacognitive ability. If individuals at CHR experience self-referential metacognition as aversive, this may increase motivation to avoid interrogating prior beliefs, contributing to global problems with belief updating. Thus, the current findings may provide a common link tying together basic self-fragmentation, narrative self-referential metacognition, and global problems with belief updating in individuals at CHR.

In the present study, CHR individuals with low positive self-beliefs also appeared to have decreased functional connectivity between the left and right PCC. The PCC is a key posterior node of the CMS involved in directing attention toward internally- or externally-directed cognition, accessing autobiographical memory, and integrating multimodal information about the self from the CMS and other brain networks^34,37^. Reduced functional connectivity between the right and left PCC could plausibly accompany the processes described above. However, the left–right PCC interaction appeared to be driven by CHR participants with very low connectivity scores (see Fig. 3) and was unrelated to any potential mediators or outcomes. This finding’s theoretical and practical implications are therefore somewhat unclear based on the current study’s evidence. Interhemispheric PCC connectivity may be a valuable target for further research in the CHR syndrome.

The current study has some clinical implications. Cognitive-behavioral treatments have been suggested to target self-beliefs in psychosis^4,7,79,81–84^. The current study suggests that changes to self-beliefs in the CHR syndrome may require both interactions with the environment and reflection on those interactions. Individuals meeting CHR criteria experience more stressful or stigmatizing interactions with the environment than their normatively developing peers^85,86^. A key process for psychosocial interventions may be to support deliberate reflection on positive interactions with the environment. For instance, behavioral activation could be combined with mindfulness-based or metacognitive approaches. The emotion exposure component of the unified protocol for treatment of emotional disorders^87^ also combines these elements. The unified protocol has shown early promise in treatment for CHR, both in its original format^88^ and when enhanced with a mobile application^89^, and the current study provides further support for continued research and application of the unified protocol.

This study’s main strengths include its sample size, inclusion of a normatively developing comparison group, administration of a measure of specific positive and negative self-beliefs, and examination of multiple potential brain networks to test theoretically informed mechanisms linking internetwork connectivity to phenomenology and behavior. This study’s main limitations include its cross-sectional design, which limits causal inferences, and its reliance on a single self-report measure of self-beliefs. Future studies could include measures of other relevant self-concept processes or experimental tasks engaging self-referential processing. Similarly, we found no significant effects for negative self-beliefs. It may be that positive self-beliefs are uniquely tied to intrinsic and extrinsic self-processing. However, the HC group reported very few negative self-beliefs (see Fig. 1), and a statistical floor effect may have suppressed true associations between negative self-beliefs and functional connectivity. The BCSS was developed for use in adult schizophrenia populations, and the strong negative self-beliefs captured by the BCSS (e.g., “I am weak”) may not capture subtler changes in negative self-concept for CHR individuals and typically developing youth. Future studies may wish to employ more sensitive measures of negative self-beliefs.

In conclusion, the present study found alterations in functional connectivity between the CMS and SMN in the CHR syndrome and showed that these alterations track maladaptive self-beliefs. These results suggest mechanisms that integrate theoretical models of basic self-fragmentation, narrative self-referential metacognition, and global problems with belief updating in the development of psychosis. Treatments to enhance positive self-concept may be valuable in this population.

## Methods

### Participants

Participants were 56 help-seeking individuals classified as CHR based on the Structured Interview for Psychosis-Risk Syndromes (SIPS)^90^ and 59 matched healthy comparison (HC) youth. Participants were recruited for a study of psychosis risk in an academic research lab via newspaper, bus, and Craigslist ads, e-mail postings, and community professional referrals. Following SIPS criteria, participants were classified as CHR based on the presence of attenuated psychotic symptoms, brief psychotic experiences, or genetic risk and functional decline within the past year. Psychotic disorders were ruled out by administering the Structured Interview for DSM-IV Axis I Disorders^91^. This study’s participants were a subset of the participants in a prior study on core beliefs in CHR youth^6^, for whom resting-state fMRI data were available. All participants from that larger sample (*N* = 73 CHR and 73 HC) with complete resting-state fMRI data were included in the current study.

### Materials

CHR status was assessed by the Structured Interview for Prodromal Syndromes (SIPS)^90^ The SIPS is a clinical interview which assesses positive, negative, disorganized, and general attenuated psychotic symptoms. The SIPS is widely used in CHR research^92^. Several variables were also extracted from the SIPS to test potential mediators and outcome variables. Single items assessed perceptual abnormalities (item P4) and motor disturbances (item G3) as potential mediators. Total scores on positive and negative symptom scales assessed overall symptom severity. Social functioning was also included as an outcome variable, assessed by the Global Functioning Scale—Social^93^, a clinician-rated scale of social functioning designed and validated for use with CHR samples.

Positive and negative self-beliefs were assessed by the Brief Core Schema Scales^1^. The BCSS is a 24-item self-report scale, with items for positive or negative beliefs about the self or others (e.g., “I am valuable”, “I am weak”, “others are hostile”) rated on a 5-point scale from 0 = “I do not believe this” to 4 = “I believe this totally”. Items are grouped into positive-self, negative-self, positive-other, and negative-other subscales, with 6 items on each subscale. The current study used only the positive-self and negative-self subscales, as beliefs about others were not relevant to study hypotheses. The BCSS have been validated in adult psychosis populations^1^, and have been used increasingly in CHR research e.g., refs. ^5,7^.

### Procedures

#### Clinical assessment

Clinical interviews and self-report questionnaires were administered in person as part of a baseline assessment battery. Clinical interviews were administered by advanced graduate students trained to reliability standards of *κ* ≥ 0.80 through gold standard videos, observation, and supervised interviews.

#### MRI acquisition parameters

Subjects were instructed to rest and close their eyes during a T2*-weighted echo planar imaging functional protocol (5 min 34 s; 3.8 × 3.8 × 3.5 mm voxels; 33 slices; FOV = 240 mm; TR = 2000 ms; TE = 29 ms; FA = 75°). MR images using a standard 12-channel head coil with a 3-Tesla Siemens Tim Trio MRI Scanner (Siemens AG, Munich, Germany). For registration to Montreal Neurological Institute (MNI) template space, structural images were collected using a T1-weighted 3D magnetization sequence (0.512 mm isotropic voxels; 224 interleaved slices; Field of View [FOV] 256 mm; time to repetition [TR] 2400 ms; time to echo [TE] 2.01 ms; GRAPPA Factor 2; flip angle [FA] 8; collection orientation: sagittal plane).

#### Resting-state data processing

The FMRIB Software Library (FSL v6.0)^94^ was used to complete data processing. FSL was used to strip the skulls from images using brain extraction, high-pass filtering (100 s), and spatial smoothing (6-mm FWHM). Functional images were aligned to the Montreal Neurological Institute (MNI) 2-mm brain template. Temporal derivative regressors were calculated with artifact detection software (ART)^95–98^. Three translation and three rotation parameters were derived, as well as additional image specific confound regressors based on brain activation and framewise movement. Brain activation outliers were calculated using the mean global brain activity, i.e., the *z*-normalized mean signal across all voxels as a function of time. Outliers were defined as any frames where global mean signal exceeded 3 SD. Framewise motion measures (composite measure of total motion, or maximum voxel displacement, across translation and rotation) were used to identify motion outliers, defined as frames where the absolute value of motion exceeded 1 mm. The resultant motion regressors were entered into the model as a temporal derivative nuisance covariate at the subject level. Anatomical images were segmented into gray matter, white matter, and CSF with SPM12 to create masks for signal extraction. The Conn toolbox extracts five temporal components from the segmented cerebrospinal fluid (CSF) and white matter, which were entered as confound regressors in the subject‐level GLM. The temporal derivative of composite motion outliers (as described above) was calculated in the ART toolbox and included as a nuisance regressor. To reduce noise each subject’s anatomical image was segmented into gray matter, white matter, and CSF to reduce global signal.

#### Data analysis

Chi-squared tests (categorical variables) and two-tailed *t*-tests (continuous variables) tested group difference on demographic variables and self-beliefs.

CONN toolbox v.18.b^99^ and SPM12 (http://www.fil.ion.ucl.ac.uk/spm/software/spm12/) were used to conduct ROI-to-ROI analyses across regions in the CMS and SMN. CMS regions were the medial prefrontal cortex (combined mask of medial orbitofrontal and rostral anterior cingulate in the Desikan brain atlas) and posterior cingulate cortex. SMN regions were the primary motor cortex and primary sensory cortex (see Fig. 2). ROIs were defined in FreeSurfer^100^, which uses cortical surface landmarks to delineate cortical areas defined in the Desikan atlas^101^. A separate mask was defined for each ROI in both hemispheres, resulting in a total of 8 masks.

Connectivity between ROIs was calculated by averaging across voxel in each ROI to create an ROI level time course. The connection between regions was then calculated with Fischer-transformed bivariate coefficient by correlating each ROI time course.

ROI-to-ROI connectivity matrices were then extracted and analyzed. Group contrasts compared connectivity patterns between groups (CHR vs. HC). Then, between-group interactions tested whether the CHR group’s unusual distribution of self-beliefs was associated with unique patterns of connectivity which were not present in the HC group. Interaction effects were defined in a general linear model in which the interaction of group and self-beliefs predicted connectivity between all eight ROIs. Significant effects were defined at FDR-corrected *p* < 0.05 for seed-level two-tailed *t*-tests. Self-belief scores were grand-mean centered prior to analyses.

Subject-level ROI-to-ROI connectivity coefficients were extracted from the model and plotted to examine significant interaction effects. Interactions were plotted as LOESS regression plots and quartile effect size plots to show any potential nonlinearity in these effects. As a post hoc analysis of external validators, Pearson correlations between connectivity, self-beliefs, potential mediators (perceptual abnormalities, motor disturbances), and outcomes (attenuated positive symptoms, attenuated negative symptoms, and social functioning) were examined in the CHR group. To limit the number of correlations tested and increase their interpretability, a single PCC-SMN connectivity score was calculated for the post hoc analyses. This score was calculated as the mean of connectivity between CMS and SMN regions of interest with significant group x self-beliefs interactions (L PCC – L Sensory, L PCC – L Motor, R PCC – R Sensory, and R PCC – R Motor).

## Data Availability

The data that support the findings of this study are available from the corresponding author upon reasonable request.
